# Rapid screening of genome edited strawberry (*Fragaria ×ananassa*) regenerants using high-resolution melting analysis followed by Amplicon sequencing

**DOI:** 10.1186/s13104-026-07792-9

**Published:** 2026-04-24

**Authors:** Kaitlyn Vondracek, Man Bo Lee, Tie Liu, Seonghee Lee

**Affiliations:** 1 Gulf Coast Research and Education center, Institute of Food and Agriculture Sciences, University of Florida, Wimauma, FL 33598 USA; 2 Horticultural Sciences Department, Institute of Food and Agricultural Science, University of Florida, Gainesville, FL 32611 USA; 3 Plant Breeding Graduate Program, Institute of Food and Agricultural Sciences, University of Florida, Gainesville, FL 32611 USA; 4 Department of Plant Resources, College of Industrial Science, Kongju National University, Yesan, 32439 South Korea

**Keywords:** Genome editing, CRISPR/Cas9, *Fragaria ×ananassa*, Mutant screening, Amplicon sequencing, High-resolution melting

## Abstract

**Objective:**

Plant transformation frequently results in large quantities of regenerant plant materials which must undergo screening prior to advancement into subsequent experiments. In-depth sequencing of such quantities for detection of editing events is costly, and preliminary selection of mutant lines based on phenotypic impacts can cause significant delays. This study aimed to develop a rapid, high-throughput workflow for early detection and genotyping of CRISPR/Cas9-mediated edits in strawberry.

**Results:**

High-resolution analysis reliably identified lines containing distinct mutation profiles, and subsequent Amplicon sequencing genotyping confirmed the presence of editing events at the targeted sites, with editing efficiencies varying among subgenomes of octoploid strawberry. This workflow offers a scalable, low-cost solution for early detection and prioritization of genome-edited cultivated strawberry lines.

**Supplementary Information:**

The online version contains supplementary material available at 10.1186/s13104-026-07792-9.

## Introduction

The cultivated strawberry (*Fragaria ×ananassa*) is an economically important small fruit crop grown around the world. Owing to the complexity of its allo-octoploid genome, introgression of new genetic variation for traits of specific interest, such as disease resistance, can be challenging. The recent release of several high-quality chromosome-scale reference genome assemblies [1–5] has resulted in greater understanding of the strawberry genome and gene functions and has facilitated the development of several molecular tools to support breeding efforts [6–8]. As part of these molecular tools, application of genome editing in cultivated strawberry has also started to gain popularity for the ability to rapidly and precisely generate novel genetic variation for a given trait of interest.

Plant transformation methods often vary from explant to explant in terms of transformation and genome editing efficiency. Thus, plantlets regenerated during genome editing experiments must undergo screening to confirm the presence of the transgene as well as confirmation of mutations at the target sites. Transgene insertion can be easily confirmed via the use of polymerase chain reactions (PCR) with primers designed to target construct-specific elements, such as selection markers. Confirmation of editing events is most often performed via sequencing. However, regeneration of plant materials through tissue culture often generates numerous individual plantlets, including many chimeric individuals, making it challenging to obtain genetically uniform lines and complicating downstream analyses. Furthermore, many traits which are targeted for genome editing cannot be easily evaluated in young plants. Due to the costs associated with genotyping large populations of regenerants, it is a common approach to allow plants to grow until the targeted phenotype is observable before selecting individuals for genotyping. For traits such as fruit quality or yield, it can take months before plants are sufficiently developed to begin evaluation. Thus, development of rapid, high-throughput screening workflows may significantly benefit strawberry functional studies.

A crude DNA extraction method which enables rapid, cheap, and high-throughput DNA extraction from large populations was previously developed for strawberry seedling and leaf tissue [9]. This method is sufficient for use with PCR-based applications, such as high-resolution melting (HRM). HRM is a genotyping method which incorporates fluorescent dye into double-stranded DNA (dsDNA) amplicons before gradual denaturation [10]. Fluorescence is measured as the dye is unbound from the dsDNA and when plotted against temperature, generates a unique curve which depends on several factors [10]. HRM can detect sequence changes as small as single nucleotide indels or substitutions, with accuracy comparable to conformation sensitive capillary electrophoresis [10–11]. In strawberry, HRM has been successfully implemented for single nucleotide polymorphism (SNP) and InDel marker genotyping [6, 9]. The sensitivity of HRM also makes it a useful tool for screening genome-edited organisms [12–15]. Combined with the crude DNA extraction method, HRM can be used to efficiently screen large numbers of plantlets for mutations. Through this screening step, it’s possible to reduce the number of samples for subsequent genotyping. Amplicon sequencing (Amp-seq) is a cost-effective DNA sequencing method which makes use of next-generation sequencing (NGS) techniques to sequence PCR amplicons. Using this approach, it is possible to generate deep sequencing coverage of specific target sites, and Amp-seq has enabled efficient detection of mutations resulting from genome editing in other species [16–17].

**Fig. 1:**
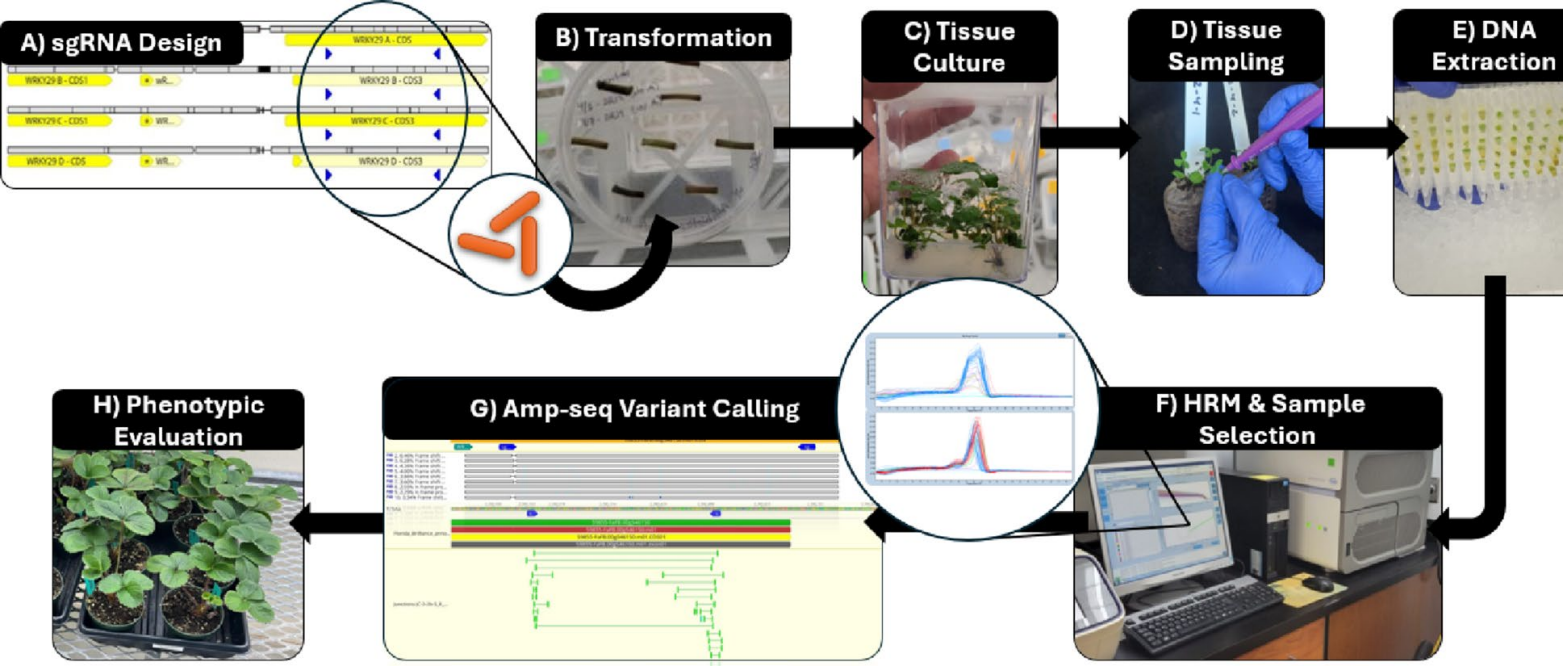
Workflow for rapid, high-throughput screening of regenerated plant materials. **A** Two sgRNAs were selected for targeting of all four homeologs of FaWRKY29. **B** ‘Brilliance’ runner tips were transformed using Agrobacterium tumefaciens strain EHA105. **C** Regenerated explants in tissue culture rooting media. **D** Sampling of young leaf tissue. **E** 96-well plate of young leaf tissue following crude DNA extraction. **F** Roche LightCycler® 480 II used for HRM analysis of crude DNA. **G** Variant calling and analysis performed using the Geneious Prime software following Amp-seq. **H** Plant lines with confirmed mutations selected for phenotypic evaluation

When combined, these methods offer the potential to quickly detect, select, and genotype mutants derived from tissue culture. While HRM and Amp-seq have previously been applied for mutant genotyping [12–18], no reports of a combined approach have been made in strawberry to the best of our knowledge. In this paper, we discuss the development of a system for rapid, high-throughput screening of genome-edited strawberry plants using rapid, crude DNA extraction followed by HRM and Amp-seq (Fig. 1).

## Materials and methods

### Design and assembly of genome editing construct

Homoeologous gene sequences of *FaWRKY29* (Fxa5Ag1276230, Fxa5Bg630890, Fxa5Cg1231820, Fxa5Dg1907300) (Supplementary File 1), a *Botrytis cinerea* susceptibility gene, were previously identified [19]. For this experiment, two single-guide RNAs (sgRNAs) were designed to target the third exon of all four homeologs of *FaWRKY29.* sgRNAs were cloned into the dual sgRNA JH4/JH19 vector system, with sgRNA-1 (5’-GGAAAGCATACCTGCTCTCA-3’) driven by the *Fragaria vesca* U6-2 promoter and sgRNA-2 (5’-GCAATGTTTCTCATTTGGCT-3’) driven by the *Arabidopsis thaliana* U6-26 promoter [20]. The completed JH19 vector was transformed into *Agrobacterium tumefaciens* strain EHA105 via electroporation.

### Plant materials

Two-week old runner tips from the commercial strawberry variety ‘Brilliance^TM^’ (FL 13.26–134 cv.) were used for agrobacterium-mediated transformation. Plants were regenerated on a series of selection media using hygromycin (2 mg/L) selection. Rooted explants were transferred to peat pellets and maintained under growth room conditions for one month before being transferred to a greenhouse at the University of Florida Gulf Coast Research and Education Center in Wimauma, Florida.

### Sample preparation and high-resolution melting analysis

Tissue sampling and DNA extraction was carried out following slight modification of the previously described method [9]. Tissue samples were collected from individual young leaves of one-month-old T_0_ plants in peat pellets using a 3 mm hole punch and maintained on ice in 96-well plates (product no. 1402–9596, USA Scientific, FL, USA). Rapid crude DNA extraction was performed immediately after tissue collection as follows: 50µL Buffer A (0.1 M NaOH, 2% v/v Tween20) was added to samples. Plates were covered with aluminum foil tape and heated over glass beads to 65℃ for 10 min. 50µL Buffer B (100mM Tris-Cl (pH 8.0), 2mM EDTA (pH 8.0)) and 100µL nuclease-free water were added, then samples were stored at 4℃ overnight to complete DNA extraction.

HRM primers were designed to generate PCR amplicons covering all homoeologous sequences of *FaWRKY29* (Supplementary Table 1). Analysis was performed with a 5 µl reaction volume using AccuStart II PCR ToughMix^®^ (Quantabio, MA, USA) in 384-well plates (product no. 1438–4690, USA Scientific, FL, USA). 0.5 µl of crude DNA extract was added to each well. HRM was run using a Roche LightCycler^®^ 480 II (Roche Life Science, Switzerland). The PCR and melting programs were carried out as previously described [9]. Analysis of HRM melt curves was carried out using the accompanying LightCycler^®^ 480 SW program. Melt curves displaying non-wild-type patterns were designated as potential mutants and a subset of lines were selected for genotyping through amplicon sequencing.

### Amplicon sequencing

PCR primers containing adapter sequences were designed to amplify a region covering both the sgRNA-1 and sgRNA-2 target sites from all homoeologous gene copies (Supplementary Table 1). Previously extracted crude DNA was used for PCR amplification. Amplicon length was confirmed using gel electrophoresis and remaining PCR products were purified using the Zymo DNA Clean & Concentrator-25 kit (Zymo Research; D4034). Samples were then submitted to Genewiz for Amplicon-EZ sequencing (Azenta Life Sciences, NJ, USA) following the posted sample submission guidelines.

Amp-seq data was processed using Minimap [21] version 2.21 and mapped to haplotype 1 of the ‘Florida Brilliance’ reference genome assembly [1]. Subsequent sequence visualization and variant analysis was carried out using Geneious Prime 2024.0.5 (https://www.geneious.com).

## Results and discussion

### Screening of edited mutants using high-resolution melting analysis

**Fig. 2:**
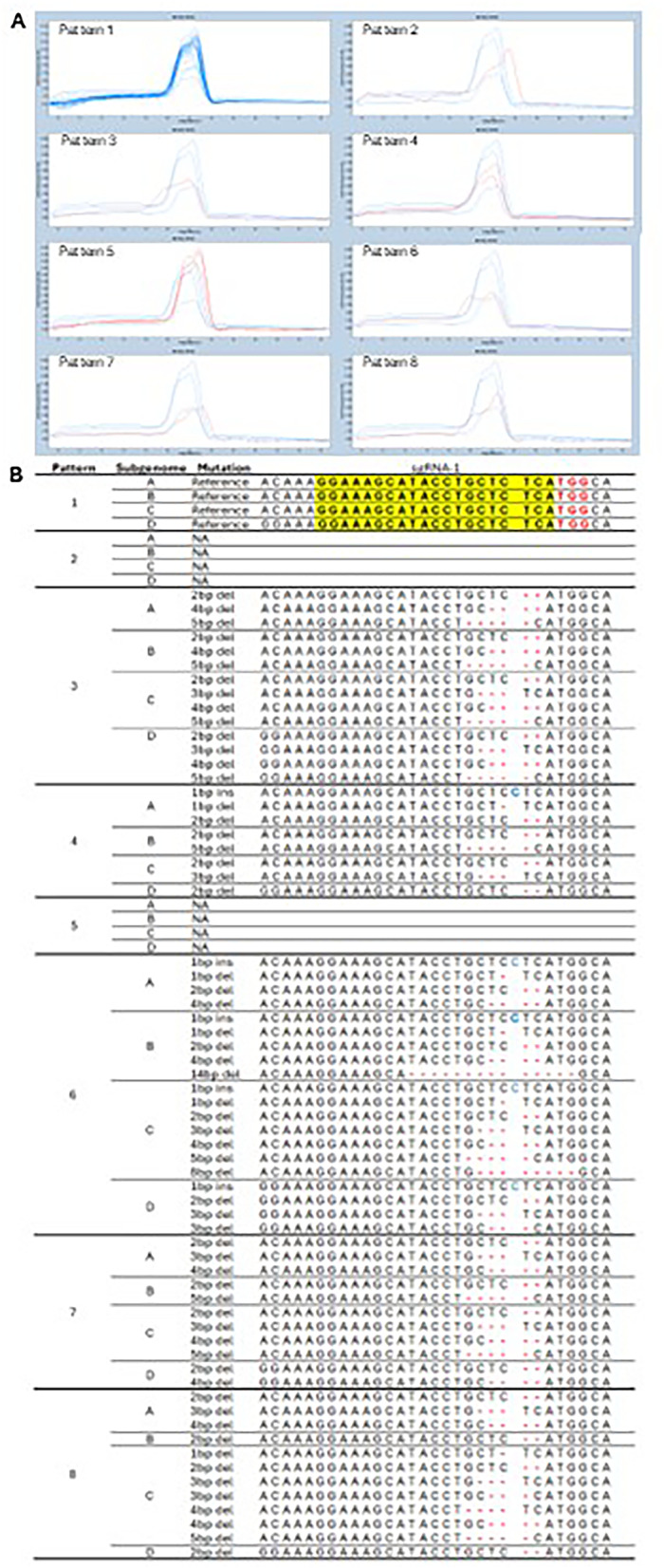
Mutant screening of the sgRNA-1 target site in *FaWRKY29*. **A** HRM screening of all regenerated lines returned eight unique melt curve profiles, including one wild-type (pattern 1) and seven mutant groups (patterns 2–7). In patterns 2–7, curves corresponding to mutant lines are shown in red and curves corresponding to wild-type controls are shown in blue. **B** Amplicon sequencing of the sgRNA-1 target site in 10 selected lines returned a wide variety of mutation compositions. The sgRNA-1 target sequence is highlighted in yellow with the corresponding PAM site indicated in red. In patterns 2–7, deleted nucleotides are indicated with red dashes and inserted nucleotides are indicated in blue

Previously, *FaWRKY29* was identified as a potential Botrytis Fruit Rot (BFR) susceptibility gene [19]. As such, sgRNAs for CRISPR/Cas9-mediated genome editing were designed to target conserved regions of all four homoeologous gene copies of *FaWRKY29* (Supplementary file 1A-D). Following Agrobacterium-mediated transformation, a total of 38 individual plants were successfully regenerated on hygromycin selection media. Analysis of HRM melt curves identified several non-wild-type curve patterns, suggesting successful regeneration of plants expressing a range of mutation profiles (Fig. 2A). Due to the high similarity of melting curve patterns for sgRNA-2 (Supplementary Fig. 1A), melting curves for the sgRNA-1 target region were used as the primary means of selecting plants for subsequent analysis. In total, ten lines displaying strong alternative sgRNA-1 profiles to the unedited controls were selected for amplicon sequencing.

### Screening of edited mutants using amplicon sequencing

Primers for generation of PCR amplicons were designed to amplify a 473 bp region from all four homoeologous copies of *FaWRKY29*. Amplicon sequencing of selected lines returned paired read depths ranging from 251,515 to 604,019 reads. Each of the 10 selected lines displayed genome editing; however, the mutant composition varied by line. A total of 7, 8, 14, and 8 unique mutations with a frequency greater than 0.5% of total reads were identified at the sgRNA-1 target site in subgenomes A, B, C, and D, respectively (Supplementary Table 2). Genome editing was lowest in subgenome C and highest in subgenome A, with an average of 40.15% and 67.92% of reads displaying non-WT sequences, respectively. Additionally, the presence of multiple mutation types within individual samples was commonly observed in the sequenced lines (Fig. 2B). No mutations with a frequency greater than 0.5% of total reads at the sgRNA-2 target site were observed (Supplementary Fig. 1B), as expected based on the high similarity to the WT pattern observed in the corresponding HRM melting curves.

### Limitations

Despite the successful, efficient identification and genotyping of edited lines, some technical and procedural limitations to this methodology must be considered. First, HRM melt curve patterns do not always shift significantly away from the WT pattern, particularly in cases of single nucleotide substitutions or small indels, which may result in a failure to identify certain edited individuals. Additionally, WT melt curves for control samples demonstrated occasional minor side-to-side shifting, suggesting that small deviations in curve position do not always indicate mutations and may result in the calling of false positives. Because the HRM steps described here are performed using crude DNA extracts, these variations may be due to inconsistent DNA quality and the presence of inhibitory secondary metabolites in addition to technical errors. While utilizing crude extracts significantly enhances throughput, it could introduce a trade-off in baseline stability that requires careful interpretation. Additionally, variant calling from Amp-seq data can be sensitive to PCR and sequencing errors, which may complicate the distinction between true low frequency edits and technical artifacts. Despite these limitations, the methods discussed herein can serve as a rapid, low-cost, and high-throughput way to screen large volumes of genome-edited plant material. Furthermore, as both HRM and Amp-seq are PCR-based, this methodology may allow for flexible and efficient analysis of any genome edited plant materials through substitution of the primers.

## Conclusions

In summary, the methodology presented in this study establishes a rapid, low-cost, and high-throughput way to screen large volumes of genome-edited plant material. By combining HRM with Amp-seq, we provide a scalable pipeline that effectively addresses and overcomes the bottleneck of mutant identification. Furthermore, as Amp-seq utilizes targeted sequencing, this methodology is highly versatile and efficient for detecting target region mutations and edited plant species. The continual release of new, high-quality reference genome assemblies and other genomic tools for *Fragaria ×ananassa* and other species has facilitated the implementation of various molecular tools, including genome editing. As the available resources for breeding and research continue to increase, so will the biotechnological approaches to crop improvement. Therefore, our findings provide an essential technical process for the development of rapid, low-cost, and high-throughput screening methods necessary for the advancement of genome editing in complex polyploid plants.

## Supplementary Information

Below is the link to the electronic supplementary material.

Supplementary Material 1. Coding DNA sequences for FaWRKY29 homeologs from subgenomes A-D. The sgRNA-1 and sgRNA-2 positions are highlighted in yellow and blue, respectively, with corresponding PAM sites indicated in red.

Supplementary Material 2.

Supplementary Material 3.

Supplementary Material 4.

## Data Availability

Plant materials used in this study are available from the University of Florida Strawberry Breeding Program located in Wimauma, Florida, USA. The sequencing datasets generated and analyzed during the current study are available from the NCBI SRA database under the accession number PRJNA1414530.

## Abbreviations

Amp-seq: Amplicon sequencing
BFR: Botrytis fruit rot
dsDNA: Double-stranded DNA
HRM: High-resolution melting
NGS: Next-generation sequencing
PCR: Polymerase chain reaction
sgRNA: Single guide RNA
SNP: Single nucleotide polymorphism
WT: Wild-type
