# Supplementary material for: Rapid screening of genome edited strawberry (Fragaria ×ananassa) regenerants using high-resolution melting analysis followed by Amplicon sequencing
Source: BMC Res Notes. 2026 Apr 24;19:245. doi: 10.1186/s13104-026-07792-9 (PMC13245021; doi:10.1186/s13104-026-07792-9)
Supplement: Supplementary file 1 — Supplementary Material 1. Coding DNA sequences for FaWRKY29 homeologs from subgenomes A-D. The sgRNA-1 and sgRNA-2 positions are highlighted in yellow and blue, respectively, with corresponding PAM sites indicated in red. [file 13104_2026_7792_MOESM1_ESM.docx]

**1A**

>Name=Fxa5Ag1276230 (FaWRKY29_A);
ATGGATACTGCTAGTAAGAGCTGGGAGCAGAAGTCACTGGTGGGGGAGAT CATTGAAGGGATGGAGCTAGCACAACAGTTAAGGCTGAGTCTGAATGCAA CATCAGCTTCATCAGAAAACAGGGAATTTTTAGTGCAGAGGATATTGACC TCATACGAAAAGGCCCTCTTACTACTGCAGTTGAGTGGCTCGCCTCAGAC ATCTCAATCGGCTGTGCTTCAGGATTTTCGTATGTCTGCCAACACAAGTC CTTGTGGTGATGACTATAACCACATGAGTTTCAAGGATCATCAGGACCTG CAAGAAATCTCCAAAAAGAGGTAACTTTAGACTATATCAAGTCATAGAAT ATGAACAAGAAAACAATGTTAATTGATAACTAATATGCTTCCTGCTACAC TTTTTGCAGAAAGAGCATGGCCAAATGGACTGACCATGTCATAAGGGTTA GCTCTGAGAATGGGATTGAAGGATCTCAGGAAGATGGCCACAGCTGGAGA AAATATGGGCAGAAAGATATTCTAGGAGCCAAATATCCTAGGTAATAAAA CAGTCTCTTTTTTAAATATTTATCTTATCCTTCTATAAAATCCATTTCAC ACATACAATCATCTGCAGCAATCTTCACTGATGCCACGTATAAAAAAGCA AGATATAGTACCCTTTCCATTACACGCACACACACCTAGTGGAAATCAGA ATCATCAATGAGAAAAGAAGTCCAACACACCTATTATGTAAAACTTGAAC TGACCCACAAAAGGAGAAACACACACCTCTTCAAAGGCATAGTTTTCACT CTGTTATGAACATATCTGATGATGAACCTTTTTGCTTATATTTGCAGAAG CTATTATAGATGCACCTTTAGGAACACACAAAGCTGCTGGGCTACAAAGC AAGTGCAGAGAGCAGATGAAGACCCAACCATATTTGAAATCACATACAAA GGAAAGCATACCTGCTCTCA**TGG**CAGCAATTCAGTTCCACCACCACCATC ACCAGAACAGCAAGAGCAAAAAAGACACAAGCGCGAGAACGTTTCTGAAC CACAGCAGTCTCAAGTGACTCAAATGAGCTTTCCAACTAATCTGAGGGTC GACACTGAGAATTTGGAAGACAAAGAGATGATGGAATCTCCATTCTCTTT TACTTCGACTTCATTTGGATGCATGCCGAGTGGAGATGACTTCCTATCTT CAATGTTTGATAATGAGAATTTCTTTGGCAATTTCTCTCAACCTTTGCTA TCTCCAGCTGCAGGCGAATCAAATTACTACTTGGCACCA**CCA**AGCCAAAT GAGAAACATTGCAGTAAATGAGCAACTTTCAGGATCTGATCTCACTGAGA TAATCTCAGCCACCAATTCCAGAACCAATTCTCCAATCCCAGACATGGAT TTCCCATTGGATCTAGTGGAACTCGACCCCAATTTCCCATTTGATACTCC

AGGATTTTTCTGA

**1B**

>Name=Fxa5Bg630890 (FaWRKY29_B);
ATGGATACTGCTAGTAAGAGCTGGGACCAGAAGTCACTGGTGGGGGAGAT CATTGAAGGGATGGAGCTAGCACAACAGTTAAGGCTGAGTCTGAATGCAA CATCAGCTTCATCAGAAAACAGGGAATTTTTAGTGCAGAGGATATTGACC TCATACGAAAAGGCCCTCTTACTACTGCAGTTGAGTGGCTCGCCTCAGAC ATCTCACGTGGATGCAAAATCGGCTGTGCTTCATGAGTCTCGTATGTCTG CCAACACAAGTCCTTGTGGTGATGACTATAACCACATGAGTTTCAAGGAT CATCAGGACCTGCCAGAAATCTCCAAAAAGAGGTAAGTTTAGATTATATC AAGCCATAGAATATGAACAAGAAAACAATGTTAATTGATAACTAATATGC TTCCTGCTACATGTTTTGCAGAAAGAGCATGGCCAAATGGACTGACCATG TCATAAGGGTTAGCTCTGAGAATGGGATTGAAGGATCTCAGGAAGATGGC CATAGCTGGAGAAAATATGGGCAGAAAGATATTCTAGGAGCCAAATATCC TAGGTAATAGAACAGTCTCTTTTTTAAATATTTATCTTATCCTTCTATAA AATCCATTTCACACATACAATCATTTGCAGCAATCTTCACTGATGCCACG TATAAAAAAGCAAGATATAGTACCCTTTCCATTACACGCACACACACAAA ACTATACATTTTAGTGGAAATCAGAATCATCAATGAGAAAAGAAGTCCAA CACACCTATTATGTAAAACTTGAACTGACCCACAAAAGGAGAAACAGACA CCTCTTCAAAGGCATAGTTTTCACTCAGTTAACACCTCTTCAAAGGCATA GTTTTCACTCAGTTATGAAGATATCTGATGATGAACCTTTTTGCTTATAT TTGCAGAAGCTATTATAGATGCACCTTTAGGAACACACAAAGCTGCTGGG CTACAAAGCAAGTGCAGAGAGCAGATGAAGACCCAACCATATTTGAAATC ACATACAAAGGAAAGCATACCTGCTCTCA**TGG**CAGCACTTCAGTTCCACC ACCACCATCACCAGAACAGCAAGAGCAAAAAAGACACAAGCGCGAGAATG TTTCTGAACCACAGCAGTCTCAAGTGACTCAAATGAGCTTTCCAACTAAT CTGAGGGTTGATACTGAGAATTTGGAAGACAAAGAGATGATGGCATCTCC ATTCTCTTTTACTTCGACTTCATTTGGATGCATGCCGAGTGGAGATGACT TCCTATCTTCAATGTTTGATAATGAGAATTTCTTTGGCAATTTCTCTCAA CCTTTGCTATCTCCAGCCGCAGGCGTATCAAATTACTACTTGGCACTA**CC A**AGCCAAATGAGAAACATTGCAGTAAATGAGCAACTTTCAGAATCTGATC TCACTGAGATAATCTCAGCCACCAATTCCAGAACCAATTCTCCAATCCCA GACATGGATTTCCCACTGGATCTAGTGGAACTCGACCCCAATTTCCCATT TGATACTCCAGGATTTTTCTGA

**1C**>Name=Fxa5Cg1231820 (FaWRKY29_C);

ATGGATACTGCTAGTAAGAGCTGGGAGCAGAAGTCACTGGTGGGGGAGAT CATTGAAGGGATGGAGCTAACACAACAGTTAAGGCTGAGTCTGAATGCAA CATCAGCTTCATCAGAAAACAGGGAATTTTTAGTGCAGAGGATATTGACC TCATACGAAAAGGCCCTCTTACTACTGCAGTTGAGTGGCTCGCCTCAGAC ATCTCTCGTGGATGCAAAATCGGCTGTGCTTCATGAGTCTCGTATGTCTG CCAACACAAGTCCTTGTGGTGATGACTATAACCACATGAGTTTCAAGGAT CATCAGGAACTGCAAGAAATCTCCAAGAAGAGGTAAGTATAGACTATATC AAGTCATAGAATATGAACAAGAAAACAATGTTAATTGATAACTAATATGC TTCCTGCTACATGTTTTGCAGAAGGAGCATGGCCAAATGGACTGATCATG TCATAAGGGTTAGCTCTGAGAATGGGATTGAAGGATCTCAGGAAGATGGC CATAGCTGGAGAAAATATGGGCAGAAAGATATTCTAGGAGCCAAATATCC TAGGTAATAAAACAGTCTCTTTTTAAAATATTTATCTTATCCTTTTCTAT AAAATCCATTTCACACATACAATCATTTGCAGCAATCTTCACTGATGCCA CGTATAAAAAAGCAAGATATAGTACCCTTTCCCTTACACGCACACACACA AAGCTATACAATTTAGTGGAAATCAGAATCATCAATGAGAAAAGAAGTCC AACACACCTATTATGTAAAACTTGAACTGACCCACAAAAGGAGAACAGAC ACCTCTTCAAAGGCATAGTTTTCACTCTGTTATGAAGATATCTGATGATG AACCTTTTTGCTTATATTTGCAGAAGCTATTATAGATGCACCTTTAGGAA CACACAAAGCTGCTGGGCTACAAAGCAAGTGCAGAGAGCAGATGAAGACC CAACCATATTTGAAATCACATACAAAGGAAAGCATACCTGCTCTCA**TGG**C AGCATTTCAGTTCCACCACCACCATCACCAGAACAGCAAGAGCAAAAAAG ACACAAGCGTGAGAACGTTTCTGAACCACAGCAGTCTCAAGTGACTCAAA TGAGCTTTCCAACTAATCTGAGGGTCGATACTGAGAATTTGGAAGACAAA GAGATGATGGCATCTCCATTCTCTTTTACTTCGACTTCATTTGGATGCAT GCCGAGTGGAGATGACTTCCTATCTTCAATGTTTGATAATGGGAAATTCT TTGGCAATTTCTCTCAACCTTCGCTATCTCCAGCCGCAGGCGAATCAAAT TACTACTTGGCACCA**CCA**AGCCAAATGAGAAACATTGCAGTAAATGAGCA ACTTTCAGAACCTGATCTCACTGAGATAATCTCAGCCACCAATTCCAGAA CCAATTCTCCAATCCCAGACATGGATTTCCGACTGGATCTAGTGGAACTC GACCCCAATTTCCCATTTGATACTCCAGGATTTTTCTGA

**1D**

> Name=Fxa5Dg1907300 (FaWRKY29_D);

ATGGATACTGCTAGTAAGAGCTGGGAGCAGAAGTCACTGGTGGGGGAGAT CATTGAAGGGATGGAGCTAGCACAACAGTTAAGGCTGAGTCTGAATGCAA CATCAGCTTCATCAGAAAACAGGGAATTTTTAGTGCAGAGGATATTGACC TCATACGAAAAGGCCCTCTTACTACTGCAGTTGAGTGGCTCGCCTCAGAC ATCTCACGTGGATGCAAAATCGGCTGTGCTTCATGAGTCTCGTATGTCTG CCAACACAAGTCCTTGTGGTGATGACTATAACCACATGAGTTTCAAGGAT CATCAGGAACTGCAAGAAATCACCAAAAAGAGGTAAGTTTAGACTATATC AAGTCATAGAATATGAACAAGAAAACAATGTTAATTGATAACTAATATGC TTCCTGCTACATGTTTTGCAGAAGGAGCATGGCCAAATGGACTGACCATG TCATAAGGGTTAGCTCTGAGAATGGGATTGAAGGATCTCAGGAAGATGGC CATAGCTGGAGAAAATATGGGCAGAAAGATATTCTAGGAGCCAAATATCC TAGGTAATAAAACAGTCTCTTTTTTAAATATTTATCTTATCCTTCTATAA AATCCATTTCACACATACAATCATTTGCAGCAATCTTCACTGATGCCACG TATAAAAAAGCAAGATATAGTACCCTTTCCATTACACGCACACACACAAA GCTATACATTTTAGTGGAAATCAGAATCATCAATGAGAAAAGAAGTCCAA CACACCTATTATGTAAAACTTTAACTGACCCACAAAAGGAGAACAGACAC CTCTTCAAAGGCATAGTTTTCACTCTGTTATGAAGATATCTGATGATGAA CCTTTTTTCTTATATTTGCAGAAGCTATTATAGATGCACCTTTAGGAACA CACAAAGCTGCTGGGCTACAAAGCAAGTGCAGAGAGCAGATGAAGACCCA ACCATATTTGAAATCACATACAAAGGAAAGCATACCTGCTCTCA**TGG**CAG CACTTCAGTTCCACCACCACCATCACCAGAACAGCAAGAGAAAAAAAGAC ATAAGCGTGAGAACGTTTCTGAACCACAGCAGTCTCAAGTGACTCAAATG AGCTTTCCAACTAATCTGAGGGTCGATACTGAGAATTTGGAAGACAAAGA GATGATGGCATCTCCATTCTCTTTTACTTCGACTTCATTTGGATGCATGC TGAGTGGAGATGACTTCCTATCTTCAATGTTTGATAATGAGAATTTCTTT GGCAATTTCTCTCAACCTTTGCTATCTCCAGCCGCAGGCGAATCAAATTA CTACTTGGCACCA**CCA**AGCCAAATGAGAAACATTGCAGTAAATGAGCAAC TTTCAGAACCTGATCTCACTGAGATAATCTCAGCCACCAATTCCAGAACC AATTCTCCAATCCCAGACATGGATTTCCCACTGGATCTAGTGGAACTCGA CCCCAATTTCCCATTTGATACTCCAGGATTTTTCTGA

**Supplementary File 1.** Coding DNA sequences for *FaWRKY29* homeologs from subgenomes A-D. The sgRNA-1 and sgRNA-2 positions are highlighted in yellow and blue, respectively, with corresponding PAM sites indicated in red.
