# Supplementary figures and images for: Rapid screening of genome edited strawberry (Fragaria ×ananassa) regenerants using high-resolution melting analysis followed by Amplicon sequencing
Source: BMC Res Notes. 2026 Apr 24;19:245. doi: 10.1186/s13104-026-07792-9 (PMC13245021; doi:10.1186/s13104-026-07792-9)

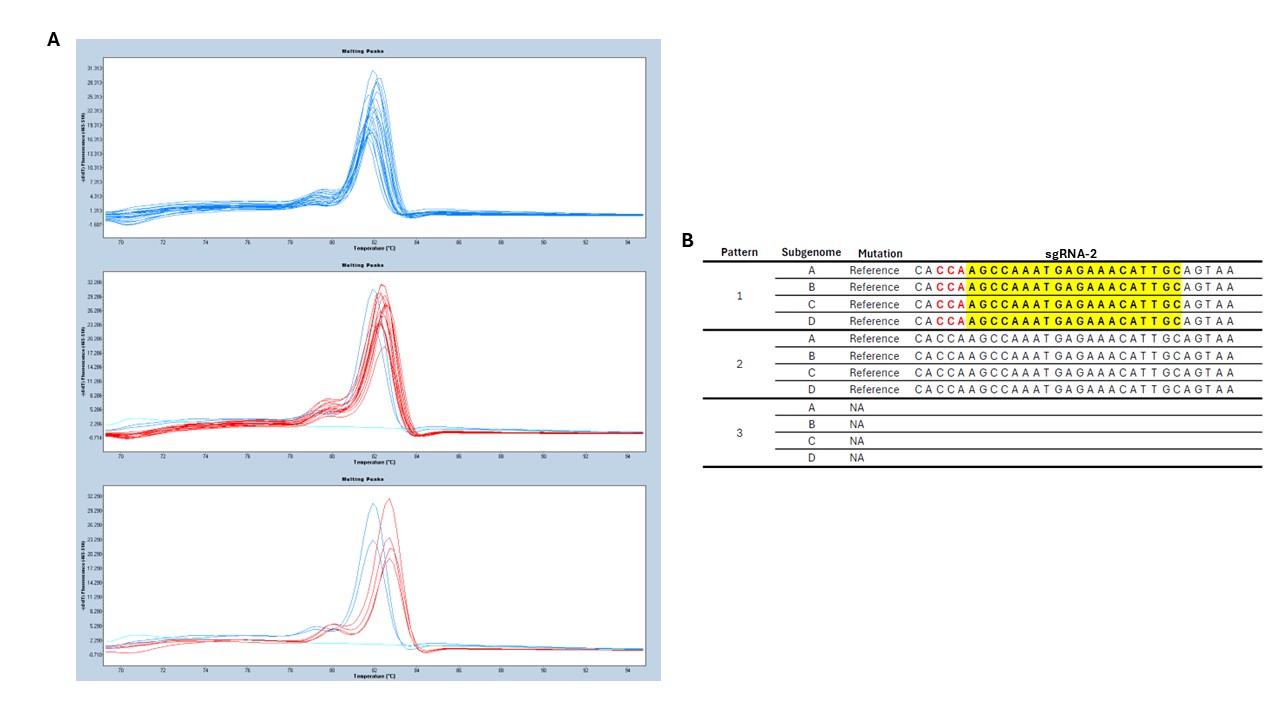

Supplement: Supplementary file 4 — Supplementary Material 4. [file 13104_2026_7792_MOESM4_ESM.jpg]
